# Psychopathology and mental health service use among youth in foster care admitted to a psychiatric inpatient unit: a 4-year retrospective controlled study

**DOI:** 10.1007/s00787-022-02104-5

**Published:** 2022-12-21

**Authors:** Mireia Solerdelcoll, Daniel Ilzarbe, Adriana Fortea, Astrid Morer, Luisa Lazaro, Gisela Sugranyes, Inmaculada Baeza

**Affiliations:** 1 Department of Child and Adolescent Psychiatry, Institute of Psychology, Psychiatry and Neuroscience, King’s College London, 16 De Crespingy Park, London, SE5 8AF UK; 2 Department of Child and Adolescent Psychiatry and Psychology, Institute of Neuroscience, Hospital Clínic de Barcelona, 2017SGR881 Barcelona, Spain; 3 Institut d’Investigacions Biomèdiques Agustí Pi i Sunyer (IDIBAPS), Barcelona, Spain; 4 Centro de Investigación Biomédica en Red de Salud Mental (CIBERSAM), Barcelona, Spain; 5 Department of Medicine, University of Barcelona, Barcelona, Spain

**Keywords:** Foster care, Psychopathology, Mental health services, Child and adolescents, Needs

## Abstract

Youth in foster care (FC) are at increased risk of poor psychosocial outcomes. The aim of this study was to assess psychopathology and mental health service use among youth living in FC who require psychiatric hospitalisation. All individuals admitted to our Children and Adolescent Inpatient Psychiatry Unit between 2014 and 2017 who were in FC were systematically reviewed. The control group was defined as all youth living with their immediate family and hospitalised in our unit throughout 2016. We identified 89 patients placed in FC and 247 controls. Socio-demographic and clinical data were retrospectively collected from computerised charts. A survival analysis of emergency department visits and readmission to the hospital was conducted. Compared to controls, the FC group presented significantly higher rates of conduct disorder (78.7% vs 14.6%; *p* < 0.001) and substance use disorder (49.4% vs 27.5%; *p* < 0.001), mainly cannabis use (34.8% vs 16.6%; *p* < 0.001); higher rates of comorbidity (96.6% vs 55.9%; *p* < 0.001) and mean number of comorbid diagnoses (3.3 ± 1.1 vs 2.3 ± 0.5; *p* < 0.001). The FC group had a higher number of emergency room visits before and after admission than controls. FC youth were also 2.77 times more likely to visit the emergency department after discharge, and in a shorter time period, than controls (*p* = 0.004). Disruptive behaviours, substance use disorder, and comorbid psychopathology were all more prevalent among FC youth than controls. Specific strategies are needed to optimize community mental health resources and address the increased use of emergency services by these youth before and after hospitalisation.

**Supplementary Information:**

The online version contains supplementary material available at 10.1007/s00787-022-02104-5.

## Introduction

Foster care (FC) is a resource that is designed to provide children and adolescents with their basic daily needs as well as the best opportunities for personal development and a stable upbringing when their immediate family is unable to care for them [1].

Nevertheless, some children’s placement trajectories end up mirroring the instability and environmental adversities they experienced before entering FC [2]. A substantial number of children entering FC experience unstable trajectories, including FC reentry and long temporary internments without reaching a permanent placement, adoption, or successful reunification. Several studies [2–4] have demonstrated a strong association between frequent FC placement moves and poor outcomes.

In recent decades, there has been a significant decrease in the number of children placed in FC [5]. However, in Spain [6], as in other certain countries [7], the number of foster placements began to rise again after 2013. A total of 47,493 children were cared for by the Spanish child welfare system in 2017, of whom 17,527 were in FC [6]. Within the region of Catalonia, according to the last report from 2018, there were 8517 children under the guardianship of the Directorate-General for the Care of Children and Teenagers (DGAIA), of whom 4800 were living in FC [8, 9]. The growing pressure on the Spanish child welfare system is largely linked to an increase in the arrival of unaccompanied migrant minors.

In light of the growing socioeconomic crisis and psychological impact surrounding the COVID-19 pandemic, child psychosocial needs have increased [10]. Evidence from previous health emergencies indicates that existing child protection risks are exacerbated, and new ones emerge, as a result not only of the epidemic but also of the socioeconomic impact of the associated control measures [11]. Thus, it is expected that the number of children at risk of family separation and in need of alternative care will increase as a result of the impact of the pandemic on families’ capacity to care [12].

Several studies have examined the childhood-to-adulthood developmental trajectories of institutionalised children [13–16], reporting higher rates of mental health conditions in children in institutional care than children not involved with the child welfare system. Longitudinal studies of foster children have revealed that they have a higher risk of being victims of any type of maltreatment, a higher risk of low educational achievement and unemployment [2, 5, 17] and higher rates of psychotropic medication prescriptions [18–20]. Accordingly, the Lancet Group Commission has advocated for global reform of the current approach to the care of separated children through the progressive replacement of institutional care with family-based care, including extended kinship networks, adoption, and stable, high-quality fostering [21]. Data from the National Survey of Child and Adolescent Well-being (NSCAW) [22] revealed a significant gap between high levels of need for mental health treatment and low rates of service use among youth involved in child welfare systems.

Although there is long-standing evidence of increased psychopathological complexity and greater needs for mental health services [23, 24] in youth placed in FC compared to both socially disadvantaged children living at home and non-welfare children [25, 26], most epidemiological studies have focused on nonclinical paediatric populations, with relatively few studies analysing the extent of mental health burden among clinical samples [27, 28]. The paucity of studies that have assessed these issues in hospitalised children raises the need to address this topic to provide new insights and review the current provision of treatment and care to this particularly vulnerable population.

The present study aimed to compare clinical characteristics and mental health service use between youth placed in FC vs. youth living at home with biological parents or other caregivers with no out-of-home placement history and who were admitted to an acute psychiatric unit. We hypothesized that: (i) children in FC would be more frequently diagnosed with behavioural disorders and would have a higher rate of comorbid disorders than the control group; and (ii) children in FC would re-consult more often, and in a shorter period of time, to the emergency department, and would have a higher rate of readmission and require readmission sooner than controls.

## Methods

### Sample

All patients under the age of 18, living in FC and admitted to the Child and Adolescent Inpatient Psychiatric ward of the Hospital Clinic de Barcelona between January 2014 and December 2017 were systematically reviewed. Analyses were restricted to patients currently placed in FC during the hospitalsiation period. Only the first hospitalisation during this period, for those admitted more than once, was considered for the analysis. A non-foster care control group of youth living at home was selected. Data from all patients under age 18 and living with their immediate family, regardless of family structure, parents’ marital status or whether the family was adoptive or biological, who were admitted to the Child and Adolescent Inpatient Psychiatric ward of the Hospital Clinic de Barcelona between January 2016 and December 2016 were systematically and retrospectively reviewed.

This study was approved by the Ethics Committee of the Hospital Clinic de Barcelona (HCB/2018/0111). Since this is a retrospective observational study, no direct or indirect risks were expected because no intervention was carried out in the sample.

### Procedures and assessments

Computerised clinical charts from a total of 1504 admitted patients were anonymously and retrospectively reviewed. A data abstraction form was created, records were hand-searched for pertinent information, and data were anonymously entered into a clinical electronic database. Socio-demographic and clinical data, including the child's race/ethnicity, age, gender, history of peer-bullying, child maltreatment (physical, sexual, or psychological abuse, or neglect) and pharmacologic treatment after discharge from hospitalisation were registered. Information about family history of mental disorders (including a diagnosis of severe mental illness or substance abuse) and FC trajectory (number of FC placements, age at first placement, family foster care, unaccompanied migrant minors) obtained from patients and/or their caregivers was supplemented with information from charts and primary psychiatric care providers.

Youth in FC usually experience some emergency and short-term FC placement before entering a comprehensively assessed long-term FC placement. A single short-term placement may therefore not necessarily represent instability, but a number of short-term placements succeeding one another, or long-term placements in temporary residential resources, suggest that the child’s needs may not have been fully met [2]. For the current analysis, the total number of placement changes was recorded after beginning long-term FC.

The reasons that led to admission were based on the clinicians’ judgment at the time of admission assessment extracted from the electronic records. At discharge, the diagnoses were made by consultant psychiatrists using the Diagnostic and Statistical Manual of Mental Disorders, Fifth Edition (DSM-5) criteria [29]. We also identified children and adolescents at ultra-high risk for psychosis; a completed description of the inclusion criteria of this subsample can be found in Dolz et al. [30]. This information was systematically recorded for every hospitalised patient.

With regards to service use and clinical characteristics, the following data were recorded: referral unit at admission and discharge, number of psychiatric admissions, length of hospital stay, number of visits to the emergency department, and the main reason for admission. Information about the time to the first emergency department visit, and the time elapsed before readmission following discharge from the inpatient unit, were collected as an indicator of recurrence or risk of recurrence*.*

### Statistical analysis

Descriptive statistical analyses were conducted in Stata IC 13.1 using t test for quantitative data and chi-square or Fisher’s exact test, when needed, for qualitative variables. A survival analysis was conducted to model the time before subsequent consultation in the emergency department and the time before hospital readmission during a follow-up period, which was recorded until December 2017. Being in FC was considered a predictive variable in Cox proportional hazards models, which included Hazard Ratios (HR). A multilevel mixed-effects linear regression model was built, where group, time, and group-by-time interaction were included as fixed effects and “individual” as a random effect, so as to evaluate the change in the number of visits to the emergency room before and after admission. To make both groups comparable in terms of follow-up time, we conducted analyses of the time to next visit to the emergency department or readmission to hospital, and number of visits before and after admission, in the subsample of subjects admitted to the inpatient unit during 2016. Finally, the analyses were repeated comparing hospitalisation and follow-up variables between groups, excluding youth with eating disorders. This was done because patients with eating disorders as a group may have specific characteristics that could potentially influence some of the observed differences between FC and controls (see supplemental material).

## Results

### Socio-demographic characteristics

Eighty-nine subjects were identified as being in FC (58.4% females), and the control group consisted of 247 youth (60.7% females). Table 1 shows the socio-demographic characteristics of both groups. There was a small yet statistically significant difference in age (15.2 ± 1.8 vs. 14.5 ± 2.2 years old; *p* = 0.003) and country of origin (44.9% vs. 17.8%; *p* < 0.001) between groups. The FC group presented higher rates of maltreatment for all types of victimisation reported (40% vs. 6%; *p* < 0.001). Family history was available for 77 cases and 226 controls (90.2% of the sample). First- and second-degree relatives of the patients in FC presented higher rates of mental disorders than control subjects (*p* = 0.003). There were significant differences in the prescribed pharmacological treatment plan after admission (see Table 3), whereby almost all individuals in FC were prescribed psychotropic medications (98.9%) and second-generation antipsychotics (95.5%) at discharge. On average, these subjects received 1.9 (range 1–4) psychotropic medications.

**Table 1:** Sociodemographic and clinical characteristics of youth in foster care and controls

	Foster care (*n* = 89)	Controls (*n* = 247)	*p*
Age, years (mean ± SD [range])	14.5 ± 2.2 [7.4–18.0]	15.2 ± 1.8 [9.4–17.9]	0.003*
Sex, female, *n* (%)	52 (58.4%)	152 (61.0%)	0.665
Ethnicity/race, white, *n* (%)	49 (55.1%)	205 (82.3%)	< 0.001*
Family history, *n* (%)			
Any axis I disorder	61 (79.2%)	138 (60.5%)	0.003*
Severe mental illness^a^	33 (42.9%)	113 (49.6%)	0.309
Substance use disorders	42 (54.6%)	53 (23.3%)	< 0.001*
Intellectual disability	5 (6.5%)	5 (2.2%)	0.067
History of maltreatment^b^, *n* (%)	32 (36.0%)	14 (6.0%)	< 0.001*
Bullying/peer victimization, *n* (%)	3 (3.4%)	15 (6.1%)	< 0.001*
Hospitalisation, days	15.8 ± 9.2 [2–51]	20.2 ± 18.0 [2–164]	0.027*
No. of readmissions, *n* (%)^c^	15 (48.4%)	73 (29.6%)	0.034*
Re-consultation to ED, *n* (%)^c^	25 (80.7%)	99 (40.1%)	< 0.001*
No. of visits to ED before admission	2.8 ± 3 [0–14]	1.7 ± 2 [0–10]	< 0.001*
Time to first visit to ED, days^d^	81.3 ± 88.6 [3–322]	161.9 ± 130.0 [2–531]	0.004*
Time to next admission, days^e^	109.4 ± 76.3 [5–315]	167.2 ± 115.8 [8–427]	0.068

*SD* standard deviation, *ED* emergency department

^a^Comprises any first- and second-degree relatives with a psychiatric disorder (including mood disorders, psychotic disorders, anxiety disorders and behaviour disorders). Family history was available for 77 foster care cases and 226 controls

^b^‘Any maltreatment’ composite created by combining all forms of reported severe maltreatment (physical, sexual, or psychological abuse, and/or neglect)

^c^Subanalysis including subjects admitted in the year 2016 (*n* = 278)

^d^Duration based on number of days leading to the first visit at emergency department after discharge within subjects admitted in the year 2016 (*n* = 124)

^e^Duration based on number of days to leading to first inpatient readmission after discharge within subjects admitted in the year 2016 (*n* = 88)

*p < 0.05

FC trajectories are detailed in Table 2, showing that children were an average age of 12.6 years (SD = 3.4; range 3.5–17.5) at the time of their first placement. Almost half (47%) experienced at least one placement movement during follow-up. Six (6.7%) adolescents were unaccompanied migrants, all of whom were males aged 14–17 years and originally from Morocco.

**Table 2:** Foster care placement variables (*N* = 89)

Characteristics	Frequency
First-time entry age, years (mean ± SD [range])	12.6 ± 3.4 [3.5–17.5]
Total time in FC, years (mean ± SD [range])	2.8 ± 2.7 [0.5–14.5]
Number of FC placements, mean ± SD [range]	1.6 ± 0.7 [1–3]
Foster family^a^, *n* (%)	24 (267)
Unaccompanied migrant children, *n* (%)	6 (6.7)

*SD* standard deviation, *FC* foster care

^a^Foster family refers to formal nonrelative foster care

### Psychopathological characteristics

As shown in Table 3, the main reason for being admitted to the inpatient unit were disruptive behaviour in FC (69.7% vs. 15.0%; *p* < 0.001), and mood symptoms in the control group (13.5% vs. 32.0%; *p* < 0.001). Individuals in FC presented higher rates of *DSM-5* diagnoses and comorbidity than control subjects. Conduct disorder was the most frequent diagnosis (78.7% vs 14.6%; *p* < 0.001), and drug misuse (in particular, cannabis; 34.8% vs. 16.6%; *p* < 0.001) was the most common comorbidity (49.4% vs. 27.5%; *p* < 0.001) in FC.

**Table 3:** Reasons for current admission and DSM-5 Diagnoses at discharge in youth in foster care and controls

Diagnosis, *n* (%)	Foster care (*n* = 89)	Controls (*n* = 247)	*χ*^2^	*p*
Reasons current admission				
Disruptive behaviour	62 (69.7%)	37 (15.0%)	94.13	< 0.001*
Bizarre behaviour^a^	16 (18.0%)	51 (20.6%)	0.29	0.589
Mood symptoms	12 (13.5%)	79 (32.0%)	11.34	< 0.001*
Suicidal thoughts/attempt	10 (11.2%)	42 (17.0%)	1.66	0.197
Risk taking behaviours^b^	10 (11.2%)	35 (14.2%)	0.49	0.486
Low BMI	2 (2.2%)	69 (27.9%)	24.27	< 0.001^e^*
Any psychotic disorder	24 (27.0)	47 (19.0)	2.47	0.116
Psychotic disorder NOS	19 (79.2)	31 (66.0)		0.482^e^
Schizophrenia	1 (4.2)	6 (12.8)		
Schizoaffective disorder	4 (16.7)	10 (21.3)		
Ultra-high risk for psychosis	4 (4.5)	13 (5.3)		1.0^e^
Any mood disorder	37 (41.6)	94 (38.1)	0.34	0.560
Adjustment disorder	23 (62.1)	28 (29.8)	20.62	< 0.001*
MDD	7 (18.9)	45 (47.9)		
Bipolar disorder	5 (13.5)	14 (14.8)		
ADHD	20 (13.4)	33 (22.5)	4.08	0.043*
ODD	17 (19.1)	11 (4.5)	18.37	< 0.001*
Conduct disorder	70 (78.7)	36 (14.6)	136.38	< 0.001*
Autism spectrum disorder	10 (11.2)	33 (13.4)	0.06	0.607
Any eating disorder	5 (5.6)	63 (25.5)	16.03	< 0.001*
OCD	1 (1.1)	14 (5.7)		0.129^e^
Intellectual disability	27 (30.3)	16 (6.5)	33.37	< 0.001*
PTSD	5 (5.6)	5 (2.0)	2.92	0.087
Tourette syndrome	2 (2.3)	5 (2.0)		1.0^e^
Personality traits	6 (6.7)	5 (2.0)	4.59	0.032*
Any substance use disorder	44 (49.4)	68 (27.5)	14.13	< 0.001*
Cannabis	31 (34.8)	41 (16.6)	12.91	< 0.001*
Alcohol	17 (19.1)	20 (8.1)	8.08	0.004*
Nicotine	29 (32.6)	54 (21.9)	4.04	0.044*
Comorbidity	86 (96.6)	138 (55.9)	48.91	< 0.001*
Number of diagnoses^c^	3.3 ± 1.1 [2–6]	2.3 ± 0.5 [2–4]		< 0.001*
Any medication after discharge	88 (98.9)	220 (89.1)	80.34	< 0.001*
Any antipsychotic^d^	85 (95.5)	186 (75.4)	17.11	< 0.001*
SG-LAIAs	22 (24.7)	14 (5.7)	34.82	< 0.001*
Antidepressants	26 (29.2)	120 (48.6)	9.98	0.002*
Mood stabilizers	20 (22.5)	37 (15.0)	2.61	0.106
ADHD medication	10 (11.2)	16 (6.5)	2.07	0.150

*Psychotic Disorder NOS* psychotic disorder no otherwise specified, *MDD* major depression disorder, *ADHD* attention-deficit/hyperactivity disorder, *ODD* oppositional defiant disorder, *OCD* obsessive–compulsive disorder, *PTSD* post-traumatic Stress disorder, *SG-LAIAs* second generation long-acting injectable antipsychotics

^a^Bizarre behaviour related to psychotic symptoms

^b^Risk-taking behaviours include substance drug abuse, risky sexual behaviours and self-harm

^c^Number of diagnoses among those with comorbidity

^d^Includes oral and/or long-acting injectable antipsychotic

^e^Fisher’s exact test

*p < 0.05

Most patients (96.6% vs. 55.9; *p* < 0.001) presented comorbidities (the mean number of comorbid diagnoses was 3; range 1–6). Both groups presented a high prevalence of mood disorder, yet they differed in terms of the main diagnosis (adjustment disorder in FC vs. major depressive disorder in controls; 62.2% and 47.9%, respectively). Individuals in FC experienced substantially higher rates of intellectual disability than controls (30.3% vs. 6.5%; *p* < 0.001). On the other hand, foster children were less likely to have an eating disorder compared with controls (5.6% vs. 25.5%; *p* < 0.001).

### Mental health services use

**Fig. 1:**
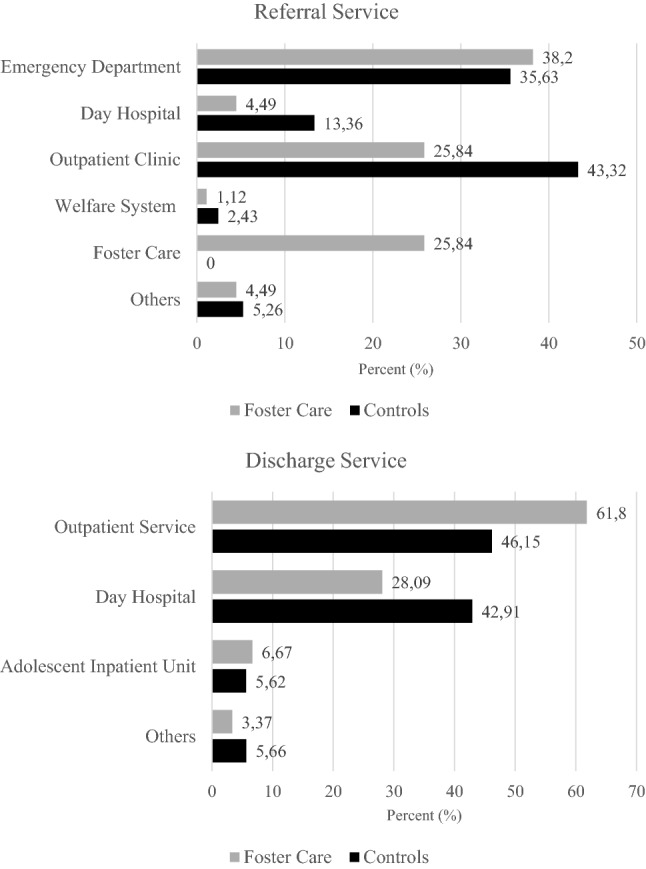
Source of referral to inpatient unit and mental health service at discharge. Others include: private healthcare service, child welfare and mental health agencies outside the Catalan health system, and therapeutic foster care unit

Figure 1 shows the rates of mental health service utilisation by youth in FC in comparison with the control group. The most prevalent referral service to the inpatient unit was the emergency department (38.2% vs. 35.6%, respectively), followed by a child and adolescent mental health outpatient clinics and the foster care providers themselves. In contrast, child and adolescent mental health outpatient clinics were the most frequent referrers among controls (25.8% vs. 43.3%, respectively). There were no significant differences between groups in terms of the discharge service; most patients were discharged to child and adolescent mental health outpatient clinics (61.8% vs. 46.2%, respectively). With regards to the length of hospitalisation (see Table 1), the duration of admission was shorter for FC patients (*p* = 0.027), and individuals in FC used the emergency department before admission more frequently than control subjects (2.8 vs. 1.7; *p* < 0.001).

### Follow-up

**Fig. 2:**
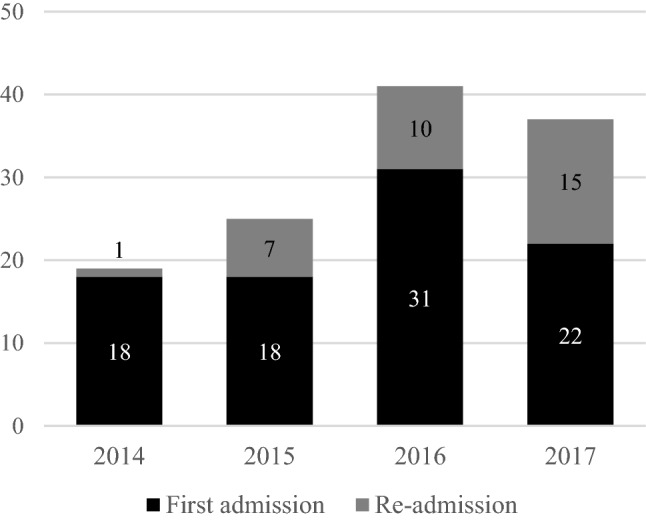
Frequencies of admissions of youth in foster care by the year

**Fig. 3:**
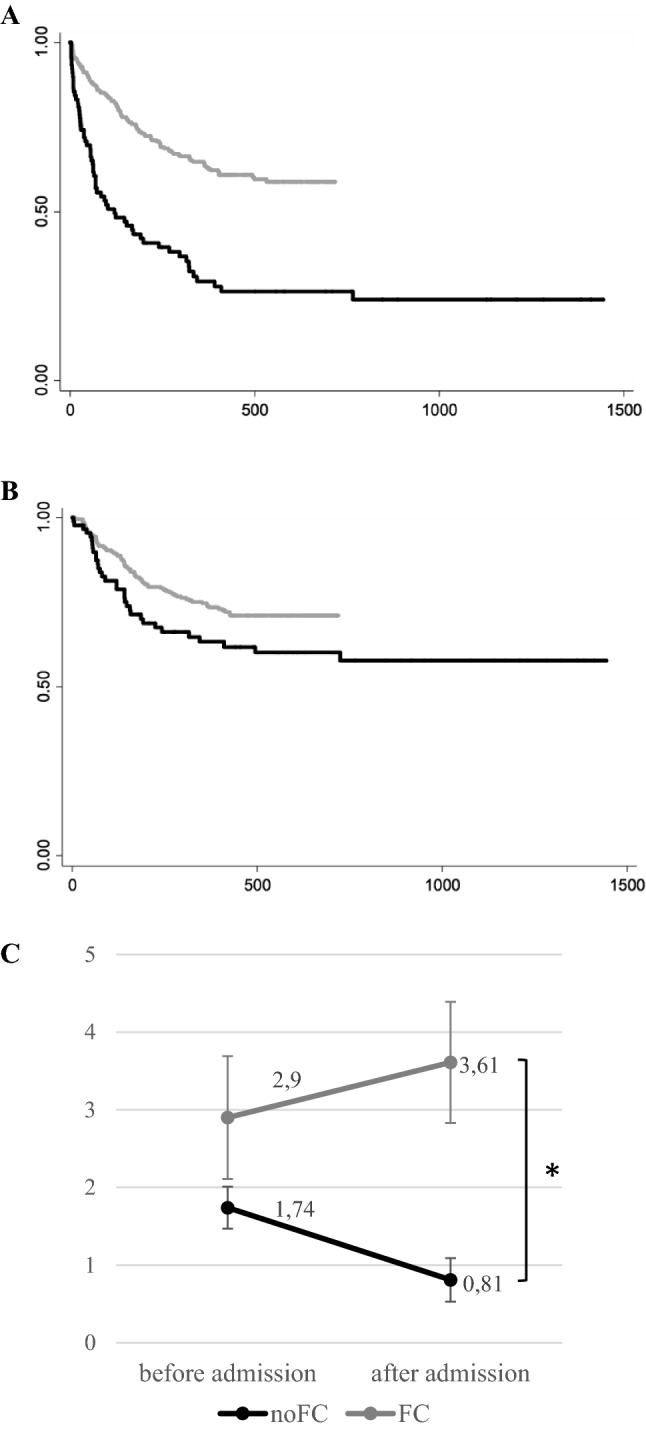
Kaplan–Meier Survival Graph for time until (**A**) first visit to the psychiatric emergency department after discharge (days) and (**B**) hospital readmission after discharge (days), comparing foster care and control group; and evolution of the number of visits to the psychiatric emergency department before and after admission for both groups (**C**)^a^. ^a^Subanalysis including subjects admitted during 2016 (*n* = 278); *p < 0.05

In recent years, the number of foster children admitted to our psychiatric inpatient unit has increased (see Fig. 2). There were significant differences between individuals in FC and controls during the follow-up period (January 2014 - December 2017) in terms of the use of psychiatric emergency services and readmissions to the inpatient ward. The FC group was likely to visit the emergency department after discharge 2.77 times more often (95%CI HR [2.00–3.82]) and sooner after discharge (81 days vs. 162 days; *p* = 0.004) than controls. In addition, individuals in FC were readmitted 1.47 times more frequently than controls (95%CI HR [0.97–2.23]), and sooner after discharge (109 days vs. 167 days; *p* = 0.07) (see Table 1, and Fig. 3a and b), a difference that was significant at a trend level. These outcomes remained constant when comorbidity was added to the Cox proportional hazards models.

Regarding the number of visits to the emergency department before and after admission, there was a significant group by time interaction (*p* < 0.001) that was due to fewer visits by control subjects after admission (*β* =− 0.93; *p* < 0.001), while there were no differences in the FC group (*β* = 0.71; *p* = 0.101). Visits to the emergency department by individuals in FC were more frequent than by controls both before and after discharge (*p* < 0.001) (see Fig. 3c).

## Discussion

To the best of our knowledge, this is the first study to perform a comparison of clinical complexity and use of mental health services among hospitalised youth who have been placed in FC and their counterparts living at home with their primary caregiver.

The main results from this study were that, compared with subjects who were never placed in FC, youth living in long-term placements had (1) a higher prevalence of mental health disorders and comorbidity, specifically disruptive behaviour disorders and drug misuse; and (2) a significantly higher use of psychiatric emergency services before and after admission, along with a significantly shorter period of time until the first visit to the psychiatric emergency department after discharge.

### Clinical characteristics

Consistent with previous studies reporting an association between looked-after children and poor mental health outcomes [17, 31, 32], this study indicates that youth in FC experienced higher rates of externalising and substance-related disorders, as well as increased rates of comorbidity and mean number of comorbid diagnoses. We found a higher prevalence of both externalising and internalising disorders in our FC group compared with other national surveys [27, 31]. This is likely due to sample selection since hospitalisation is usually required only in the most complex and severe cases.

Several clinical implications arise from these findings. First, approximately 80% of youth in FC were diagnosed with a conduct disorder, a five-fold increase compared with individuals in the control group. Children in out-of-home care represent a heterogeneous population that exhibits distinctive patterns of externalising symptoms over time, and multiple factors predict behavioural symptoms. Recognizing a socio-ecological framework, large-scale social determinants (e.g., socio-economic situation), contextual factors (e.g., placement-related factors), family factors, and child characteristics (e.g., age at first placement, socio-emotional competence, and adverse life experiences such maltreatment) shape the development of problem behaviour [33, 34]. Although family factors, such as negative and inconsistent parenting styles and caregiver mental health and/or substance use disorders, play a central role in the development of children's behaviour, and may at least partially explain such differences, factors related to foster placement (i.e., the number of previous placements and length or kind of placement) may also influence the development of problematic behaviours [35]. A longitudinal study showed that behavioural problems in foster children seemed to increase or remain stable over time during the FC placement [34]. According to studies of child welfare samples, youth in FC with conduct disorder, and especially those with comorbid attention-deficit/hyperactivity disorder (38% co-occurrence in the FC group), are more likely to engage in aggressive and delinquent behaviour and/or substance abuse in adulthood [36, 37]. Given the high associated personal and societal costs, there is a need for effective and evidence-based programmes to prevent and treat early-onset disruptive behaviour disorder in this vulnerable population [38–40]. For example, socio-emotional development, such as prosocial or self-regulation skills, moderates the relationship between out-of-home care and child behavioural outcomes and significantly improve behavioural outcomes [33]. Moreover, as we observed in our FC sample, comorbidity is more the rule than the exception, so clinicians should thoroughly assess comorbid conditions and address them appropriately. Recognizing that different factors contribute to the development of conduct disorder, interventions need to target these multiple levels. Programmes that provide foster carers with practical skills in managing child behaviour, like the Fostering Changes Programme, have been demonstrated to improve outcomes for the children in care [40, 41].

A second meaningful implication is that, in our sample, adolescents in FC had nearly twice the risk of substance misuse compared with their counterparts, and conduct disorder has been demonstrated to be the highest risk factor for substance misuse in this population, along with post-traumatic stress disorder and high levels of impulsive behaviour [42, 43]. Therefore, interventions targeting substance use should be incorporated into multimodal programmes aimed at helping these high-risk youths. Early psychoeducation interventions that provide skills for coping with aversive environments and with the potential to prevent well-established familial aggregation of substance use are valuable in reducing the burden of substance misuse [44].

Third, our findings are in keeping with those of several studies pointing to a high prevalence of mood symptoms in youth placed in FC [17, 31], with approximately two in five of all FC youth diagnosed with an affective disorder. Although there were no differences in rates of any *DSM-5* mood disorder between groups, significant differences were observed in regard to the type of diagnostic category. Compared with youth in FC, the control group had significantly higher rates of major depressive disorder and received significantly more antidepressant treatment, whereas the majority of youth in the FC sample were diagnosed with an adjustment disorder. This finding has implications for clinicians working with youth in alternative care. Assessment of trauma should be systematically included in clinical assessments, given that early detection and targeted psychotherapeutic interventions of stressful events have the potential to change the course of early psychopathology [45]. Furthermore, teenagers with untreated adjustment disorder are at heightened risk of developing major depression and substance use disorders [46, 47]. On the other hand, our research indicates that there is a lack of use of evidence-based assessments and interventions in clinical practice for children in FC. This overrepresentation of adjustment disorders may lead to foster youth not receiving adequate support for mood disorders, exacerbating disadvantage, and constitute a missed opportunity to improve their outcomes.

### Mental health service use

Our second objective aimed to assess the use of mental health services. Our findings are the first to report on comparative rates of psychiatric emergency department use between hospitalised youth placed in FC relative to those who are not in alternative care. Patients in FC showed substantially higher rates of psychiatric emergency department use before and after the index hospitalisation and were more likely to reconsult the emergency department during the follow-up period and in a shorter time after discharge. Moreover, youth in FC were 1.5 times more likely to be readmitted to the hospital, although the time until readmission was not significantly different between groups. Our findings also suggest that hospitalisation is not equally effective for both groups. While hospitalisation was associated with more robust and long-lasting effects in the control group, it was less likely to substantially change the pattern of psychiatric emergency department use among foster children (see Fig. 3). Adjusting models for eating disorders did not change the results (see supplemental material). In fact, the length of hospitalisation was no longer significant and differences in both the frequency and the time to readmission were statistically significant. Our findings are in line with those of Stein et al.’s study of children investigated by child welfare [48]. Given the recognition of existing deficits in mental health services and the barriers to receiving community-based treatment, the increased use of acute health services is of particular concern as it suggests that the existing mental health service model is not appropriate and that the needs of this specific population are not being fully met. Addressing these disparities in service use is an important step toward building a more evidence-based and specialist community-based mental health system, as generic services do not do justice to the complexity of these children [49]. Additionally, improving the adequacy and quality of care of current services, both in the inpatient unit and in the emergency department, should be a priority.

Outpatient clinics are the most frequently used mental health service by youth in FC, with only a small proportion requiring hospital admission [50, 51]. However, standard child mental health treatments do not appear to have the same effect on behavioural or emotional difficulties in foster youth, either because the resources do not meet their needs or because formal mental health services may be insufficient for this population [52, 53]. Our findings indicate that children in FC would benefit from attending specialist outpatient mental health programmes that can provide comprehensive therapeutic approaches that focus on the individual as well as the systems around the child [38]. Given that a subgroup of youth in FC have a complex clinical presentation with multiple comorbidities and a high demand for inpatient and outpatient psychiatric resources, intensive and multidisciplinary care units may be greatly beneficial.

Another notable finding was the high rate of prescription of psychotropic medications among the FC sample, with 98.9% of these subjects taking one or more of these medications; this is consistent with findings from other studies on out-of-home care [49, 54]. These findings indicate that psychotropic medication may be prescribed at particularly elevated rates to youth in FC with a history of hospitalisation. A high risk of these prescribing patterns has been found among youth in FC, who have complex mental health needs and a history of placement changes or hospitalisations [55]. It could be argued that youths in FC who require admission to a psychiatric unit may be more likely to have severe and complex mental health disorders requiring more use of medication than general FC samples. Nevertheless, concerns have been raised that high rates of polypharmacy and off-label use of medications such as antipsychotics may show a tendency for clinicians to rely on these prescriptions instead of providing additional psychosocial interventions and long-term support [56]. Additionally, FC children’s more tenuous connections with caregivers might also result in less advocacy for psychosocial interventions for behavioural problems, less engagement with services, and more fragmented support. The study also points out the difficulty of institutions in facilitating the provision of mental health care and treatment to these children under the same conditions as those living in the household. All stakeholders should invest in evidence-based psychosocial interventions to address behavioural needs and potentially lessen psychotropic medication use and hospitalisation rates [49].

Our findings need to be interpreted in light of several limitations. First, our sample selection focused on more severely ill youth requiring hospitalisation may limit the generalisability of the results to all youth in FC since, arguably, the more severely ill the patients are, the greater the need for medication and intervention. A second limitation is that the control group presented a significantly higher prevalence of eating disorder diagnoses, which tend to be associated with longer lengths of hospital admission [57]. Finally, the retrospective nature of the study limits our capacity to assess causal associations and evaluate other potentially relevant variables or confounders that were missing in the medical records. It is also certainly possible that we are overrepresenting the impact of foster placement on well-being because of the inability to detect all family-level risk factors or other baseline factors. Understanding whether behavioural problems diminish, persist, or increase over time, and the underlying causal relationships, will require prospective, controlled studies.

However, our study also has certain strengths. For instance, the inclusion of a control group comparable in terms of severity grants weight to our findings regarding the clinical complexity observed in youth in FC. Another strength relates to generalisability since our subjects were recruited from the Hospital Clinic de Barcelona, which is an urban, tertiary care facility, that provides services to a representative ethnic and socio-demographic mix of eligible patients.

In summary, our findings provide evidence that children in FC who require admission to a psychiatric unit are a particularly vulnerable population with a complex presentation, who show higher rates of disruptive behaviours and drug misuse than their peers. The results also indicate that FC youth may require higher rates of pharmacological interventions and greater use of the psychiatric emergency department before and after admission. Our findings highlight that the mental health needs of children and adolescents in FC could differ from those never placed out-of-home, and they reinforce the need for prevention strategies and specialist outpatient mental health programmes. Further research is needed to investigate long-term outcomes and evaluate the impact and effectiveness of such interventions.

## Supplementary Information

Below is the link to the electronic supplementary material.

Supplementary file1 (PDF 139 KB)

## Data Availability

All data generated or analyzed during this study are included in this published article.

## Declarations

### Transparency

The corresponding author (MS) affirms that the manuscript is an honest, accurate, and transparent account of the study being reported; that no important aspects of the study have been omitted; and that any discrepancies from the study as planned have been explained. The authors of this article had access to all study data, are responsible for all contents of the article, and had authority over manuscript preparation and the decision to submit the manuscript for publication
