# Supplementary material for: Psychopathology and mental health service use among youth in foster care admitted to a psychiatric inpatient unit: a 4-year retrospective controlled study
Source: Eur Child Adolesc Psychiatry. 2022 Dec 21;33(1):39–50. doi: 10.1007/s00787-022-02104-5 (PMC9768764; doi:10.1007/s00787-022-02104-5)
Supplement: Supplementary file 1 — Supplementary file1 (PDF 139 KB) [file 787_2022_2104_MOESM1_ESM.pdf]

SUPPLEMENTARY MATERIAL

Material supplementary to:

Title: **Psychopathology and Mental Health Service Use Among Youth in Foster Care Admitted to a Psychiatric Inpatient Unit: A 4-Year Retrospective Controlled Study**

Journal: European Child & Adolescent Psychiatry

Authors: Mireia Solerdelcoll[1,2], MD, Daniel Ilzarbe, MD, PhD, Adriana Fortea, MD, Astrid Morer, MD, PhD, Luisa Lazaro, MD, PhD, Gisela Sugranyes, MD, PhD, Inmaculada Baeza, MD, PhD.

[1]Department of Child and Adolescent Psychiatry, Institute of Psychiatry, Psychology & Neuroscience, King's College London, London, UK

[2]Department of Child and Adolescent Psychiatry and Psychology, Institute of Neuroscience, Hospital Clínic de Barcelona, Barcelona, Spain, 2017SGR881

[3]Department of Medicine, University of Barcelona, Barcelona, Spain

Email: mireia.solerdelcoll_arimany@kcl.ac.uk

Results on hospitalisation and follow-up variables excluding youth with eating disorders

Results excluding youth with eating disorders, due to the small prevalence in the Foster Care (FC) group and the high prevalence in the non-FC group, highlighted the severity of the FC group. Previous difference in the length of hospitalisation became non-significant once excluded the patients with eating disorder, who usually require longer admission to recover weight (see table S1). Regarding follow-up, there were still significant differences between individuals in FC and controls during the follow-up period (from January 2014 to December 2017) in terms of use of psychiatric emergency services and readmissions to the inpatient ward. The FC group was likely to visit the emergency room after discharge 2.80 times more often (95%CI HR: 1.99-3.93) and in a shorter period than controls (83 days vs. 161 days; $p = .007$). In addition, individuals in FC were readmitted 1.70 times more frequently than controls (95%CI HR: 1.10-2.64),

and now achieving statistical differences as well as in the period of time leading up to readmission (109 days vs. 185 days; p = .02) (see table S1, figure S1A and S1B). Regarding the number of visits to the emergency room before and after admission, there was a significant group by time interaction (p < .001) due to a reduction of the number of visits in controls (β=-1.01; p < .001), while there were no differences in the FC group (β=.70; p = .136). Visits to the emergency department in individuals in FC were more frequent than in controls both before and after discharge (p < .001) (see figure S1C).

Tables and figures:

Table S1. Clinical characteristics of youth in foster care and controls (excluding youth with an eating disorders).

	**Foster Care (n=84)**	**Controls (n=184)**	***p***
**Hospitalisation**, days	15.5 ± 9.2 [2-51]	17.8 ± 14.2 [2-111]	.169
**No. of readmissions, n** (%)[a]	15 (50.0%)	53 (28.8%)	.021*
**Re-consultation to ED, n** (%)[a]	24 (80.0%)	79 (42.9%)	<.001*
**No. of visits to ED before admission**	2.9 ± 3.1 [0-14]	1.9 ± 2 [0-9]	.002*
**Time to first visit to ED**, days [b]	83.6 ± 89.7 [3-322]	161.0 ± 127.6 [2-499]	.007*
**Time to next admission**, days [c]	109.4 ± 76.3 [5-315]	184.7 ± 116.5 [27-427]	.021*

[a] Subanalysis including subjects admitted at year 2016 (n = 214);
[b] Duration based on number of days leading to first visit at emergency department after discharge within subjects admitted at year 2016 (n = 103);
[c] Duration based on number of days to leading to first inpatient readmission after discharge within subjects admitted at year 2016 (n = 68);
SD = standard deviation; ED = Emergency department; *: p < .05.

Figure S1. Kaplan-Meier Survival Graph for time until (A) first visit to psychiatric emergency department and (B) hospital readmission after discharge, comparing foster care and control group; and evolution of the number of visits to the psychiatric emergency department for both groups (C) excluding youth with eating disorders

(A) Time until first visit to psychiatric emergency department after discharge (days)

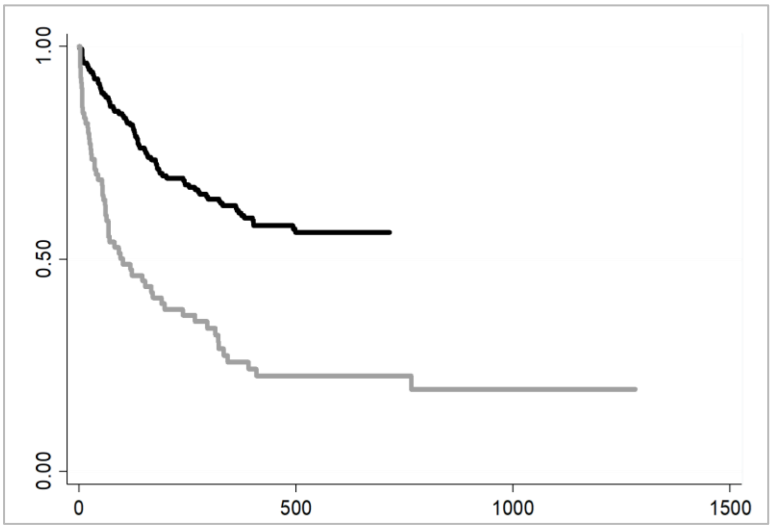

(B) Time until hospital readmission after discharge (days)

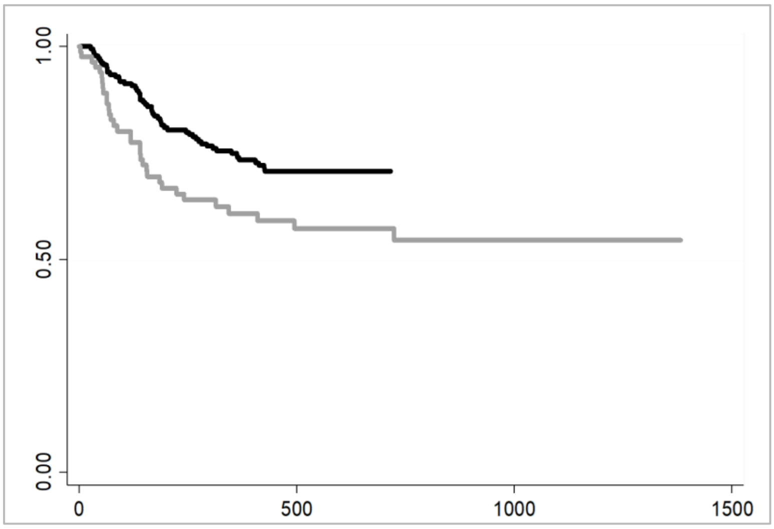

(C) Number of visits to the psychiatric emergency room before and after admission [a]

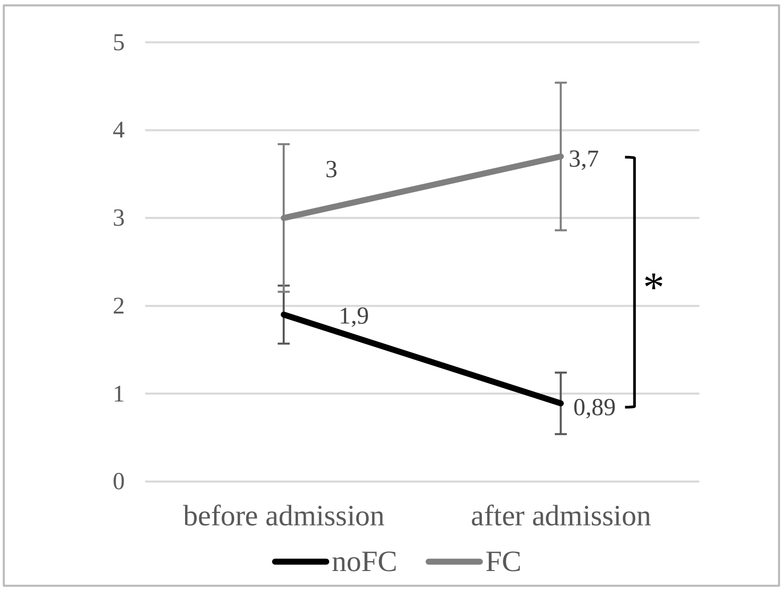

[a]: Subanalysis including subjects admitted at year 2016 (n = 214); *p < .05
